# Supplementary material for: Myc-Related Mitochondrial Activity as a Novel Target for Multiple Myeloma
Source: Cancers (Basel). 2021 Apr 1;13(7):1662. doi: 10.3390/cancers13071662 (PMC8037116; doi:10.3390/cancers13071662)

Supplementary Materials: Myc-Related Mitochondrial Activity as a Novel Target for Multiple Myeloma

Alejandra Ortiz-Ruiz[1-2†], Yanira Ruiz-Heredia[1-2†], María Luz Morales[1-2], Pedro Aguilar-Garrido[1-2], Almudena Garcia-Ortiz[1-2], Antonio Valeri[1-2], Carmen Bárcena[3], Rosa María García-Martin[3], Vanesa Garrido[1-2], Laura Moreno[1-2], Alicia Gimenez[1-2], Miguel Ángel Navarro-Aguadero[1], María Velasco-Estevez[1], Eva Lospitao[4], María Teresa Cedena[2], Santiago Barrio[1-2], Joaquín Martínez-López[1-2#], María Linares[1-2#] and Miguel Gallardo[1-2#*]

**Patient therapies**		MGUS (N=15)	SMM (N=19)	NDMM (N=27)	RRMM (N=22)
**Therapy lines before sample collection (%)**	RD	0	5,3	3,85	18,2
	VRD	0	0	0	22,7
	VMP	0	0	0	4,5
	VTD	0	0	0	4,5
	KRD	0	0	0	0
	KDD	0	0	0	0
	VBMCP	0	0	0	9,1
	Other therapies[a]	0	0	3,85	41
	No therapy	100	94,7	92,3	0
**Therapy lines following sample collection (%)**	RD	0	5,3	14,8	18,5
	VRD	0	5,3	0	18,5
	VMP	0	15,7	18,5	11,1
	VTD	0	5,3	3,7	3,7
	KRD	0	5,3	0	0
	KDD	0	0	0	0
	VBMCP	0	0	0	14,8
	Other therapies[a]	0	5,3	37,1	14,9
	No therapy	100	57,8	25,9	18,5

Table S1. Patient treatment regimens. Patient therapies and frequency of treatment according to each stage of the disease (N=123).

Abbreviations: KDD, carfilzomib daratumumab dexamethasone; KRD, carfilzomib lenalidomide dexamethasone; RD, lenalidomide dexamethasone; VBMCP, vincristine doxorubicin melphalan cyclophosphamide prednisone; VMP, bortezomib melphalan

prednisone; VRD, bortezomib lenalinomide dexamethasone; VTD, bortezomib thalidomide dexamethasone

[a]Other therapies (22): lenalidomide dexamethasone and clarithromycin; melphalan + prednisone; isatuximab + velcade + clarithromycin + dexamethasone; velcade + dexamethasone; dexamethasone + cyclophosphamide; darzalex + velcade + melphalan + prednisone; velcade + cyclophosphamide + prednisone; clinical trial CC-92480; vincristine + carmustine + doxorrubicine + dexamethasone; velcade + cyclophosphamide + dexamethasone; isatuximab; vincristine + carmustine + melphalan; ciclophosphomide + prednisone; clinical trial NKaes; pomalidomide + dexamethasone; bendamoustine + velcade + dexamethasone; darzalex + velcade + dexamethasone; clinical trial CASTOR; carfilzomib + dexamethasone; carfilzomib + cyclophosphamide + dexamethasone; elutuzumab + thalidomide + dexamethasone; clinical trial POMDEFIL; aplidin; clinical trial MK3475.

**Antibodies**	**MW kDa**	**Used dilution**	**Source**
OXPHOS	54, 48, 29, 22, 18	1:1000	Abcam
c-Myc	60	1:1000	Abcam
B-actin	45	1:1000	Cell Signaling
Goat polyclonal antibody anti-mouse	-	1:1000	Dako
Goat polyclonal antibody anti-rabbit	-	1:1000	Dako

Table S2. Antibodies used for western blotting.

A

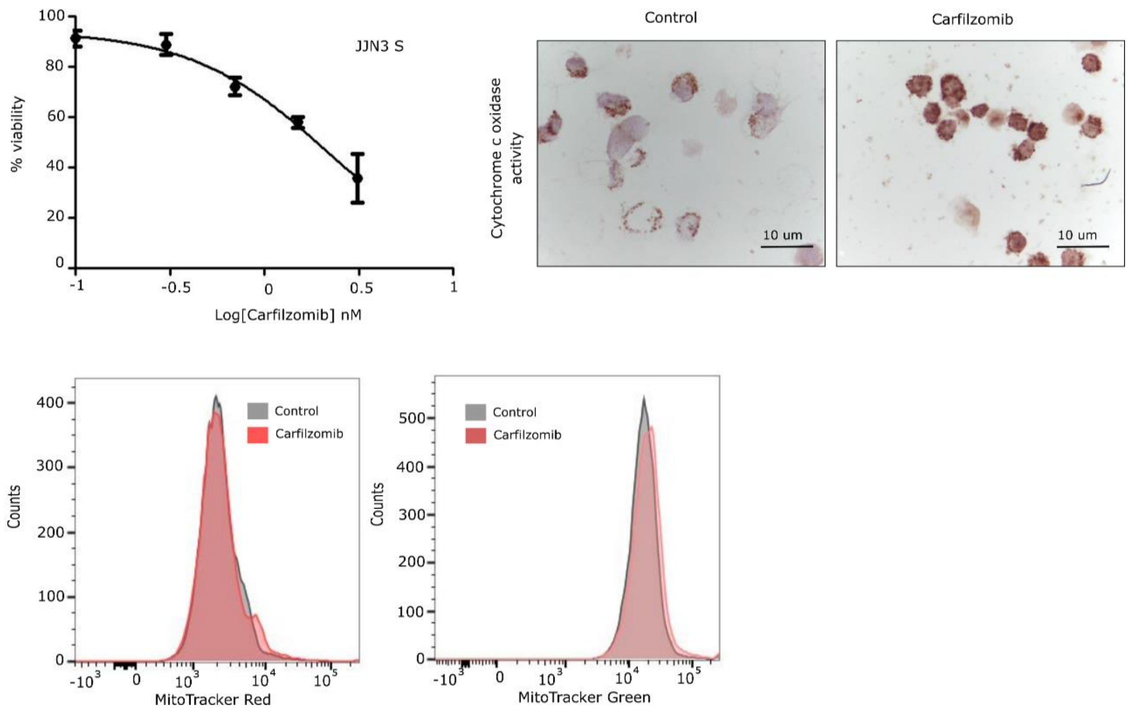

Figure S1. Contribution of carfilzomib treatment in mitochondrial activity features. **A** Study of carfilzomib cytotoxic effects in JJN3 S cells. Analysis of mitochondrial activity by histoenzymatic analysis of COX activity in JJN3 S cells treated with 0.1% DMSO (control) or 1.5 nM carfilzomib; and flow cytometry analysis of MitoTracker Red (left, mitochondrial membrane potential) and green (right, mitochondrial mass) in JJN3 S cells with 0.1% DMSO (control, grey) or 1.5 nM carfilzomib (red) (n=3).

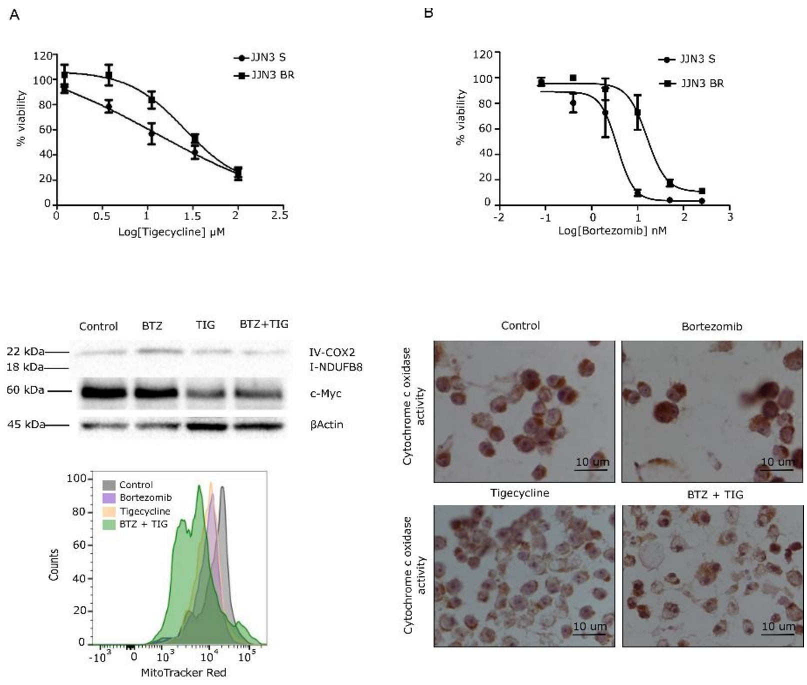

Figure S2. Effect of tigecycline and the combination with bortezomib on JJN3 BR cells. **A** Comparison of tigecycline activity in JJN3 S and JJN3 BR cells. **B** Comparison of bortezomib activity in JJN3 S and JJN3 BR cells. **C** Molecular validation of mitochondrial inhibition of JJN3 BR cells treated with 3.7 μM tigecycline, 2 nM bortezomib and their combination (n=3): by WB of respiratory chain complexes I and IV and c-Myc; by HE-COX; and by Multiparametric Flow Cytometry (MFC) analysis of MitoTracker Red (mitochondrial membrane potential). All of the drug treatments were for 48 h; n= 3.

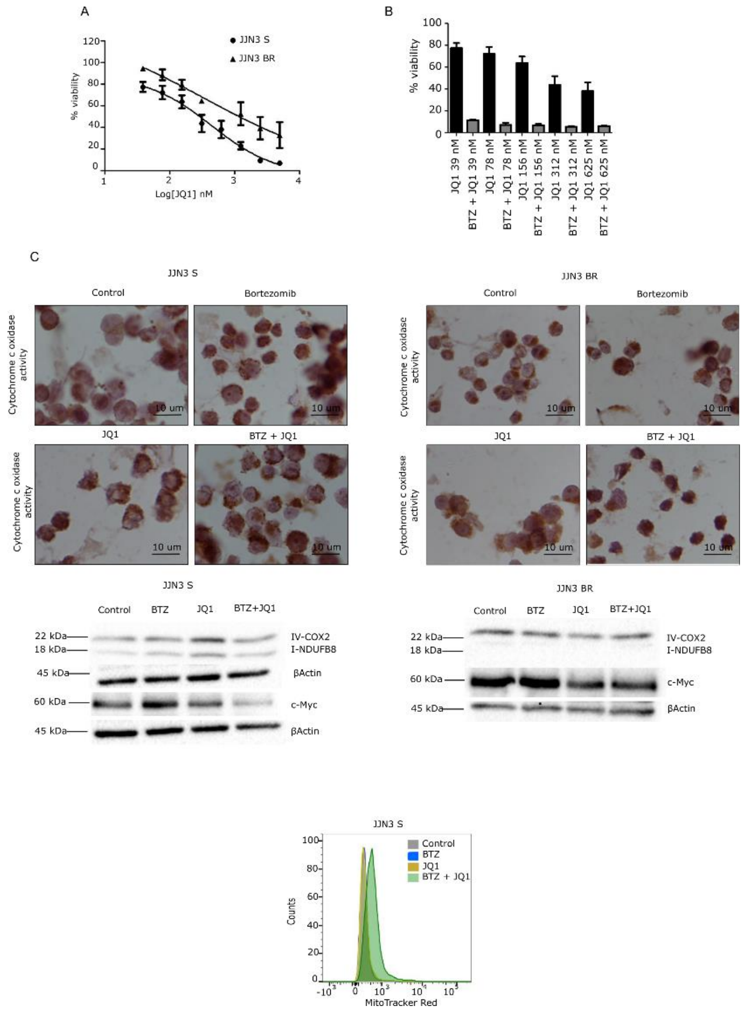

Figure S3. Validation of tigecycline mode of action with JQ1. JQ1 and the combination with bortezomib inhibits cancer cell growth and c-Myc expression in the JJN3 cell line **A** Activity of 78 nM JQ1 on JJN3 S and JJN3 BR cell viability (n=3). **B** JQ1 alone or in combination with bortezomib **C** Molecular analysis of the JQ1-mediated effect on mitochondrial activity in JJN3 S and JJN3 BR cells by MitoTracker Red flow cytometry, by HE-COX, and by WB of respiratory chain complexes I and IV and c-Myc. All of the drug treatments were for 48 h; n= 3.

Figure 3C

OXPHOS
Left 4 lines used in manuscript (inverted image)
Loading order:
Combination - Tigecycline - Bortezomib - Control

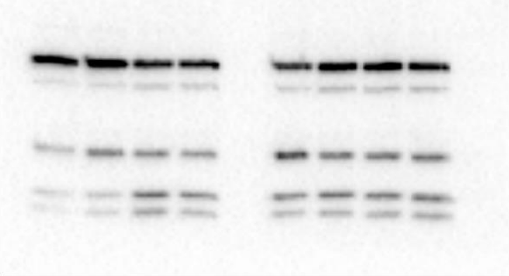

c-Myc
Right 4 lines used in manuscript
Loading order:
Control - Bortezomib - Tigecycline - Combination

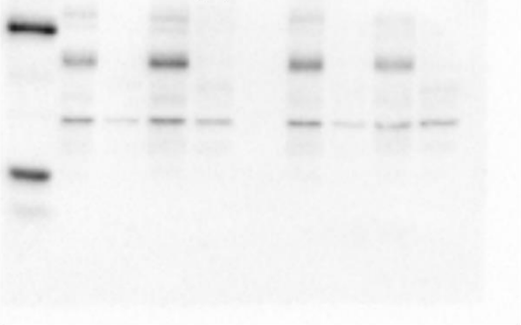

GAPDH
Left 4 lines used in manuscript (inverted image)
Loading order:
Combination - Tigecycline - Bortezomib - Control

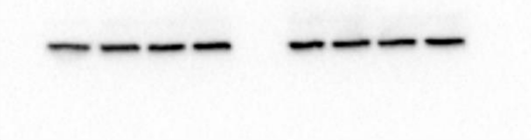

B-actin
Right 4 lines used in manuscript
Loading order:
Control - Bortezomib - Tigecycline - Combination

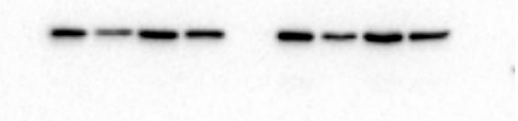

Figure S2B

OXPHOS
Loading order:
Control - Bortezomib - Tigecycline - Combination

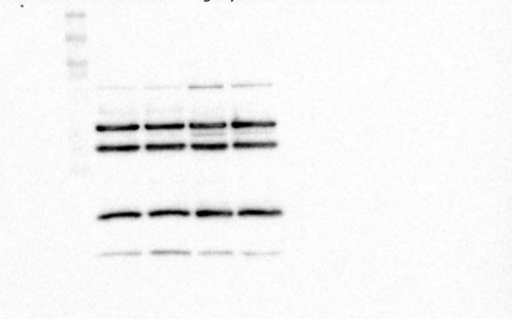

c-Myc
4 top lines used in manuscript

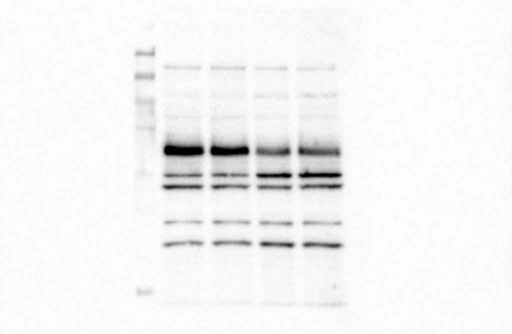

B-actin
4 intermediate lines used in manuscript

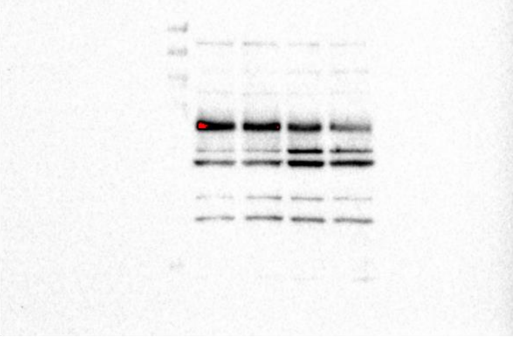

Figure S3C

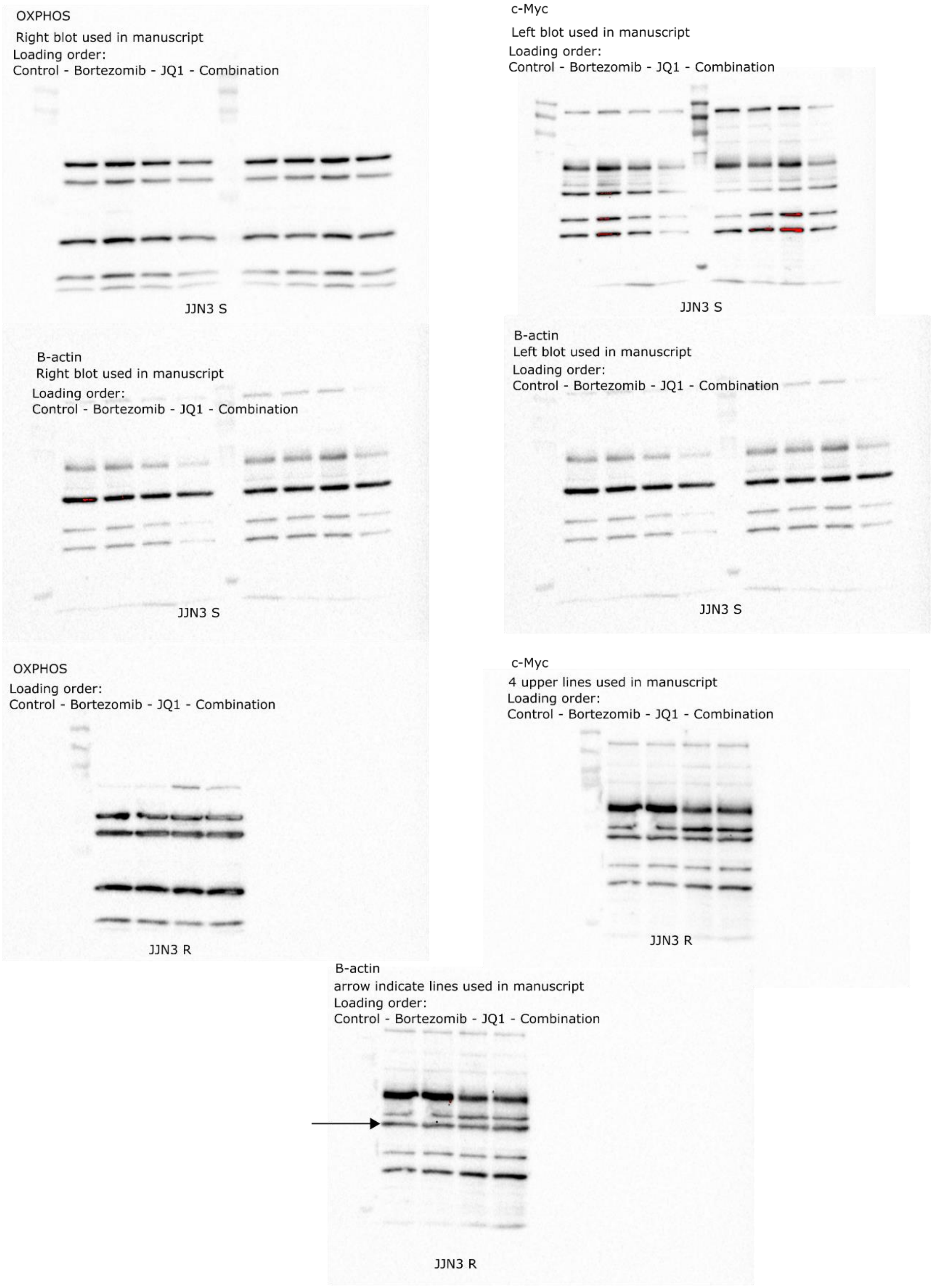

Figure S4: uncropped western blot.

A

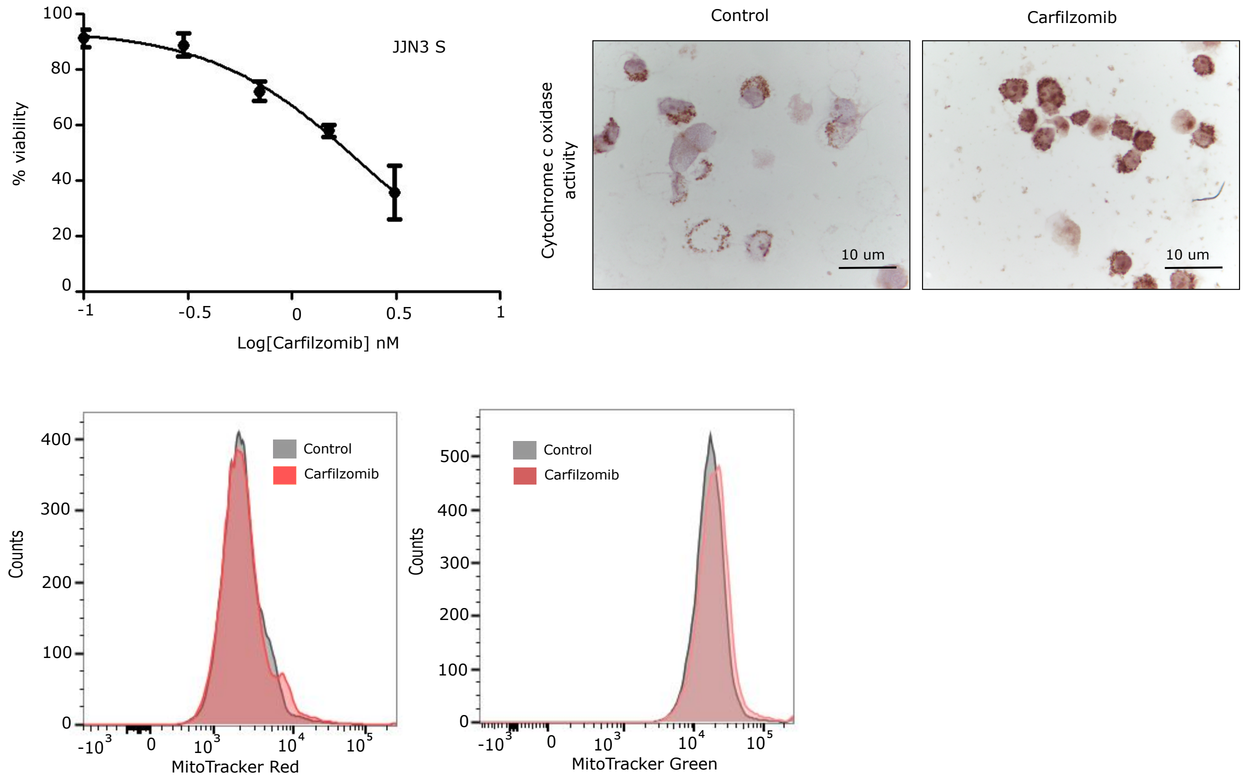
JJN3 S
% viability
100
80
60
40
20
0
-1
-0.5
0
0.5
1
Log[Carfilzomib] nM
Control
Carfilzomib
Cytochrome c oxidase activity
10 um
10 um
Control
Carfilzomib
Counts
400
300
200
100
0
-10^3
0
10^3
10^4
10^5
MitoTracker Red
Control
Carfilzomib
Counts
500
400
300
200
100
0
-10^3
0
10^3
10^4
10^5
MitoTracker Green

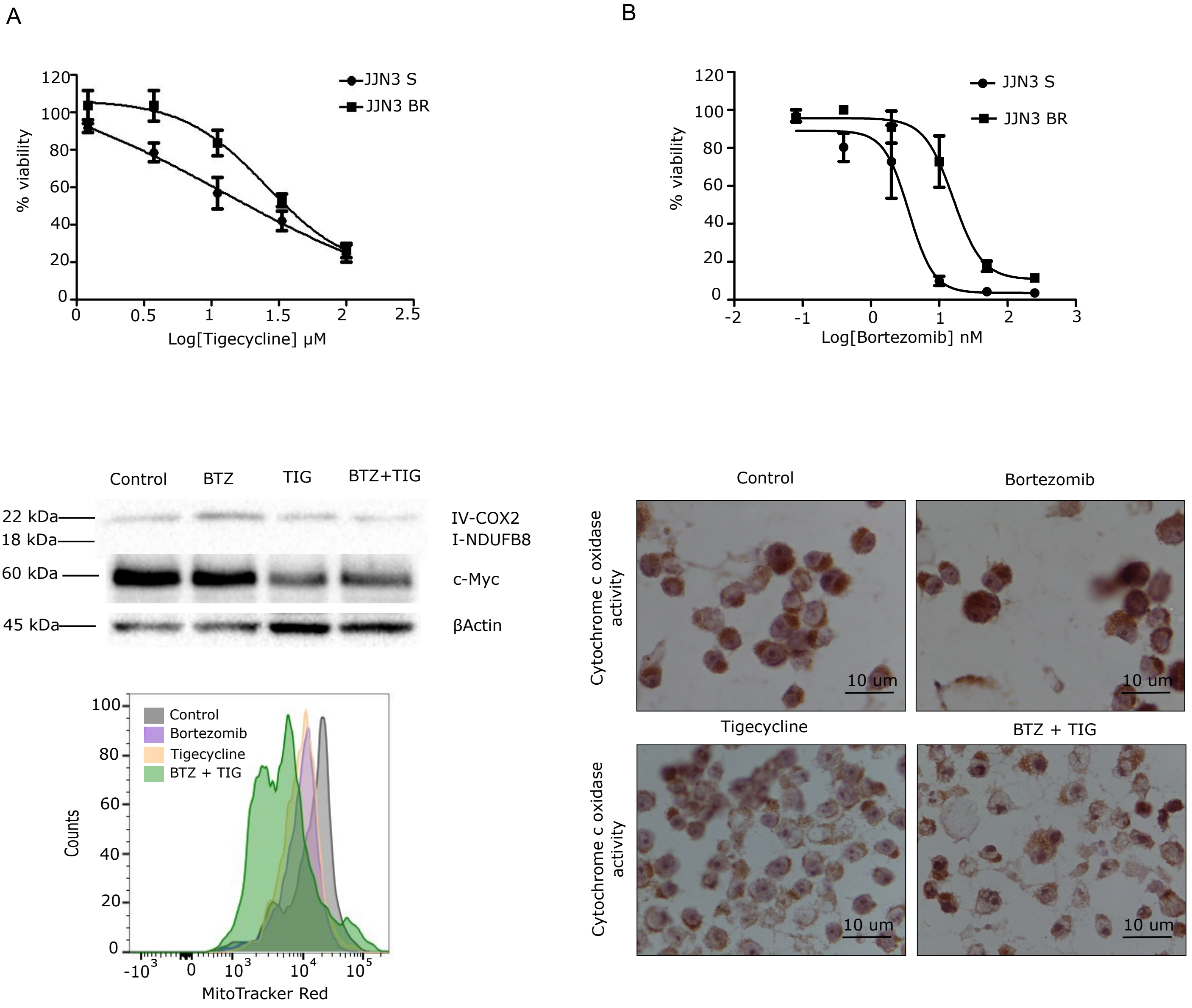
A
JJN3 S
JJN3 BR
% viability
Log[Tigecycline] μM
B
JJN3 S
JJN3 BR
% viability
Log[Bortezomib] nM
Control
BTZ
TIG
BTZ+TIG
22 kDa
18 kDa
60 kDa
45 kDa
IV-COX2
I-NDUFB8
c-Myc
βActin
Control
Bortezomib
Tigecycline
BTZ + TIG
Counts
MitoTracker Red
Control
Bortezomib
Tigecycline
BTZ + TIG
Cytochrome c oxidase activity
10 um

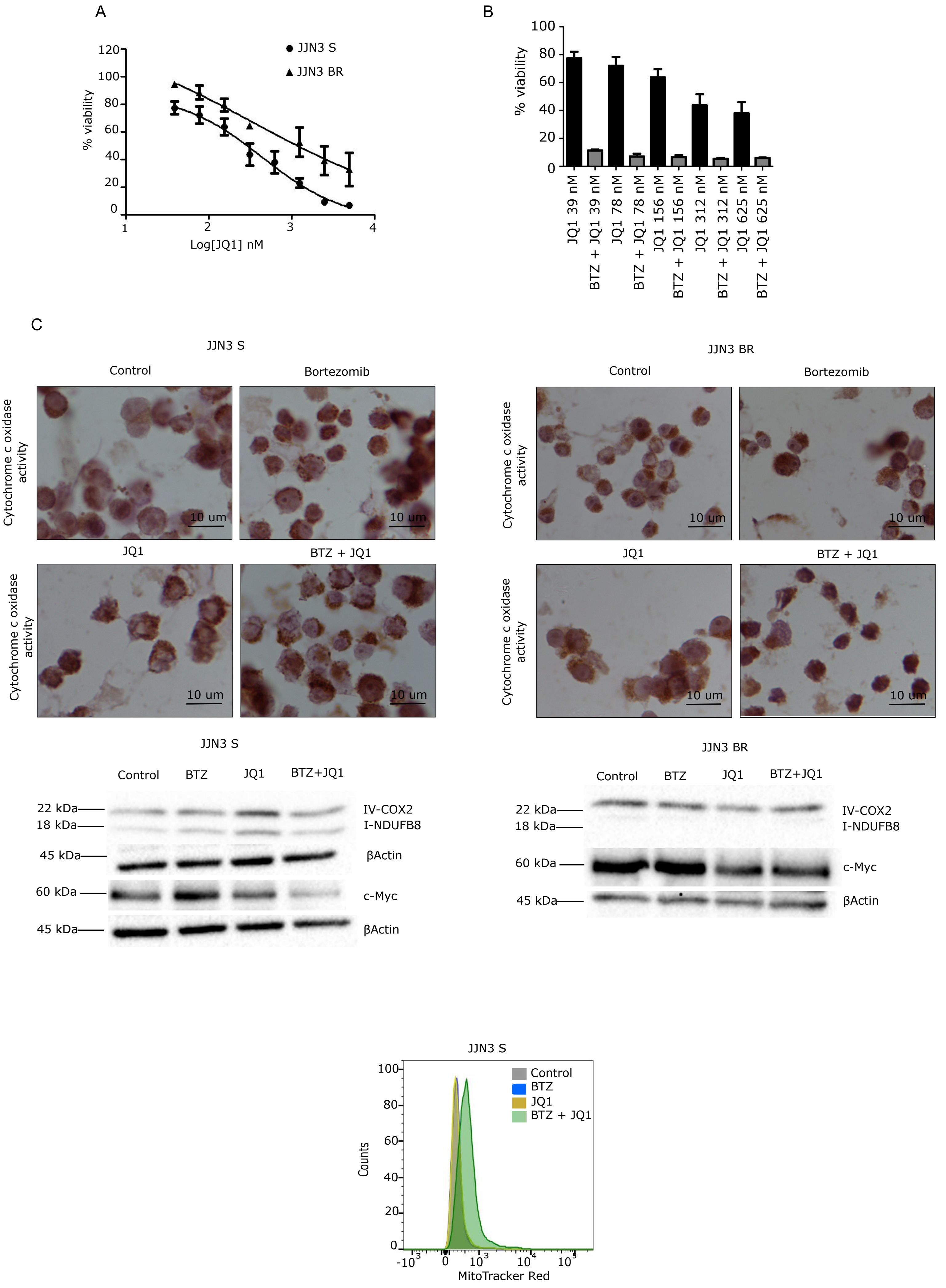
A
JJN3 S
JJN3 BR
% viability
Log[JQ1] nM
B
% viability
JQ1 39 nM
BTZ + JQ1 39 nM
JQ1 78 nM
BTZ + JQ1 78 nM
JQ1 156 nM
BTZ + JQ1 156 nM
JQ1 312 nM
BTZ + JQ1 312 nM
JQ1 625 nM
BTZ + JQ1 625 nM
C
JJN3 S
Control
Bortezomib
JQ1
BTZ + JQ1
Cytochrome c oxidase activity
10 um
JJN3 BR
Control
Bortezomib
JQ1
BTZ + JQ1
Cytochrome c oxidase activity
10 um
JJN3 S
Control
BTZ
JQ1
BTZ+JQ1
22 kDa
18 kDa
45 kDa
60 kDa
45 kDa
IV-COX2
I-NDUFB8
βActin
c-Myc
βActin
JJN3 BR
Control
BTZ
JQ1
BTZ+JQ1
22 kDa
18 kDa
60 kDa
45 kDa
IV-COX2
I-NDUFB8
c-Myc
βActin
JJN3 S
Control
BTZ
JQ1
BTZ + JQ1
Counts
MitoTracker Red

Figure 3C

OXPHOS

Left 4 lines used in manuscript (inverted image)

Loading order:
Combination - Tigecycline - Bortezomib - Control

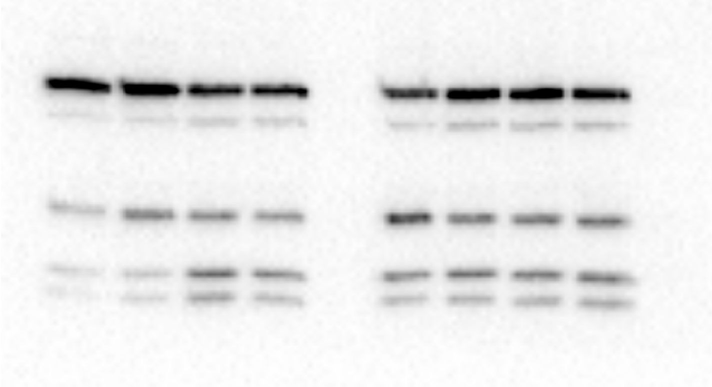

c-Myc

Right 4 lines used in manuscript

Loading order:
Control - Bortezomib - Tigecycline - Combination

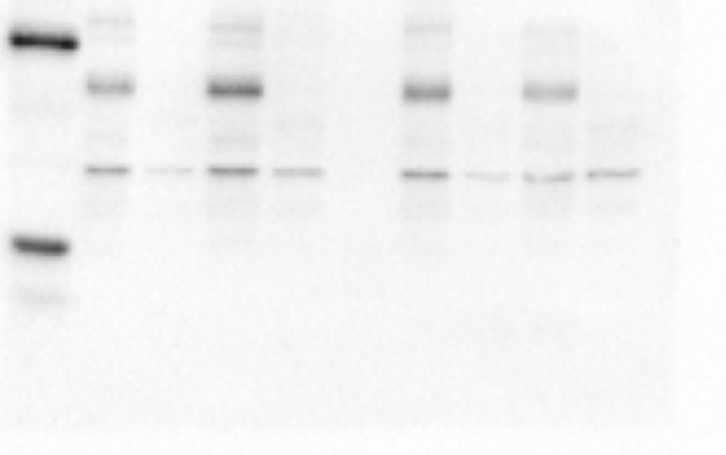

GAPDH

Left 4 lines used in manuscript (inverted image)

Loading order:
Combination - Tigecycline - Bortezomib - Control

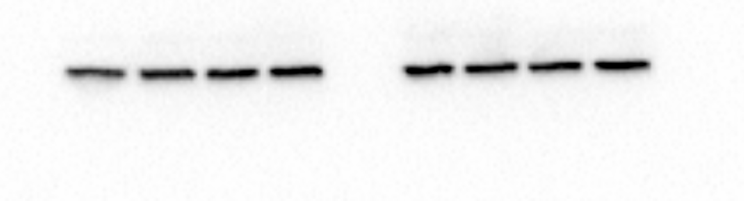

B-actin

Right 4 lines used in manuscript

Loading order:
Control - Bortezomib - Tigecycline - Combination

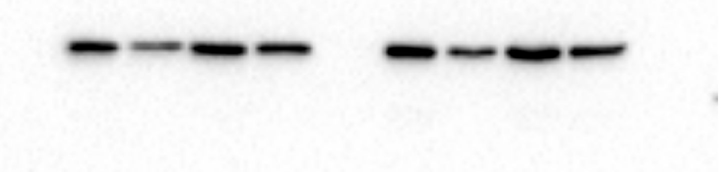

Figure S2B

OXPHOS

Loading order:
Control - Bortezomib - Tigecycline - Combination

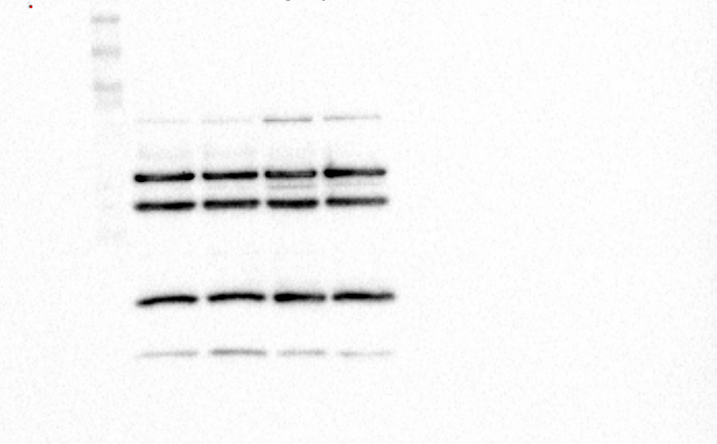

c-Myc

4 top lines used in manuscript

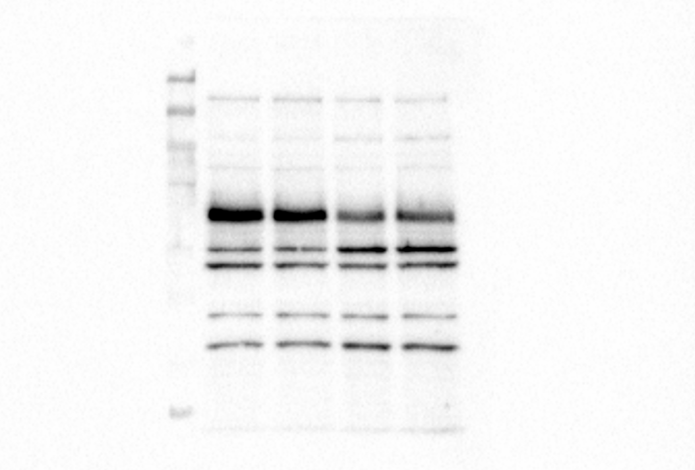

B-actin

4 intermediate lines used in manuscript

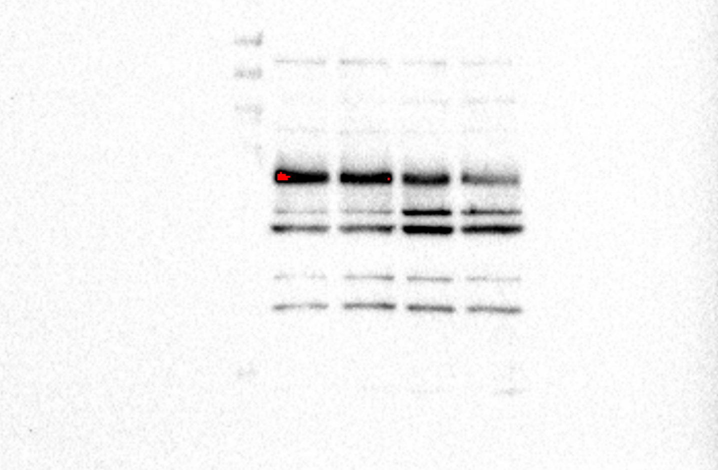

Figure S3C

OXPHOS

Right blot used in manuscript

Loading order:

Control - Bortezomib - JQ1 - Combination

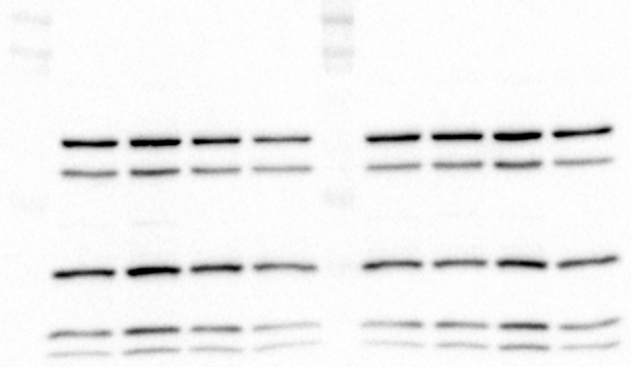

JJN3 S

c-Myc

Left blot used in manuscript

Loading order:

Control - Bortezomib - JQ1 - Combination

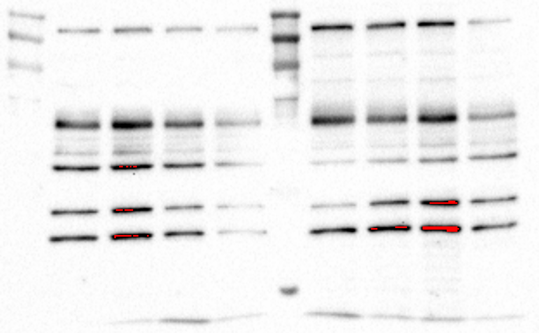

JJN3 S

B-actin

Right blot used in manuscript

Loading order:

Control - Bortezomib - JQ1 - Combination

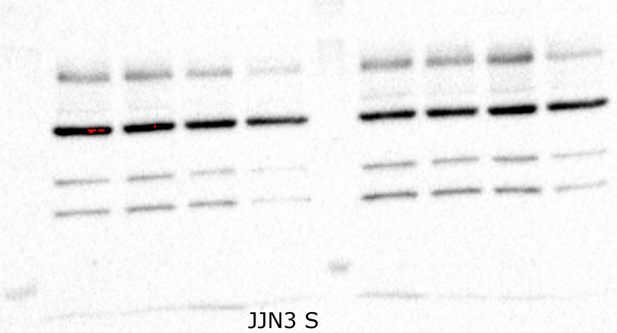

JJN3 S

B-actin

Left blot used in manuscript

Loading order:

Control - Bortezomib - JQ1 - Combination

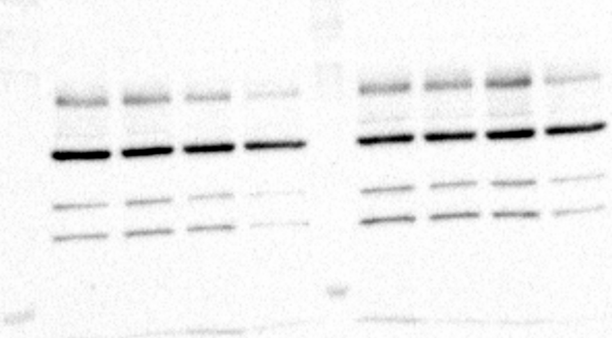

JJN3 S

OXPHOS

Loading order:

Control - Bortezomib - JQ1 - Combination

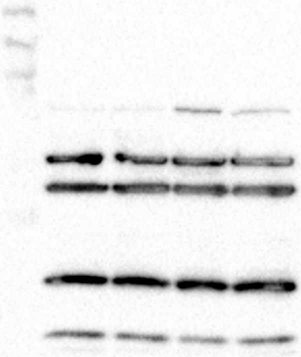

JJN3 R

c-Myc

4 upper lines used in manuscript

Loading order:

Control - Bortezomib - JQ1 - Combination

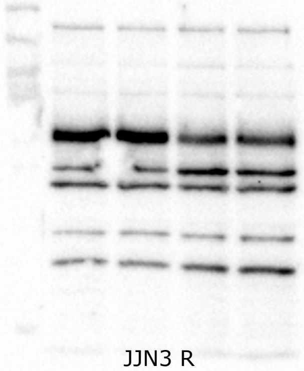

JJN3 R

B-actin

arrow indicate lines used in manuscript

Loading order:

Control - Bortezomib - JQ1 - Combination

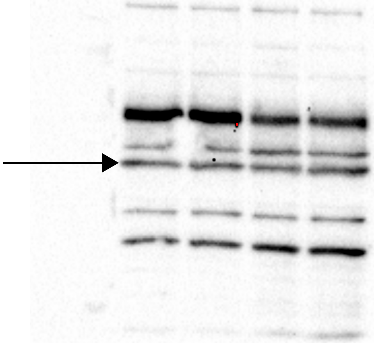

JJN3 R

Figure S4A

OXPHOS

Right blot used in manuscript, lanes 7 and 8

Loading order:
Control - Daratumumab

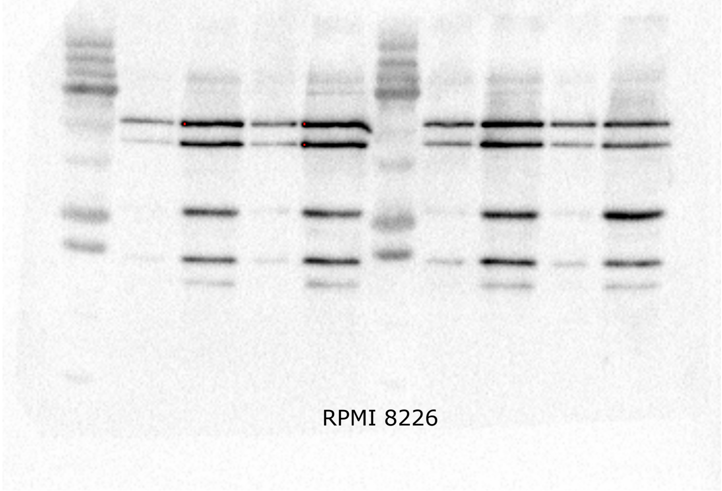

c-Myc

Right blot used in manuscript, lanes 7 and 8

Loading order:
Control - Daratumumab

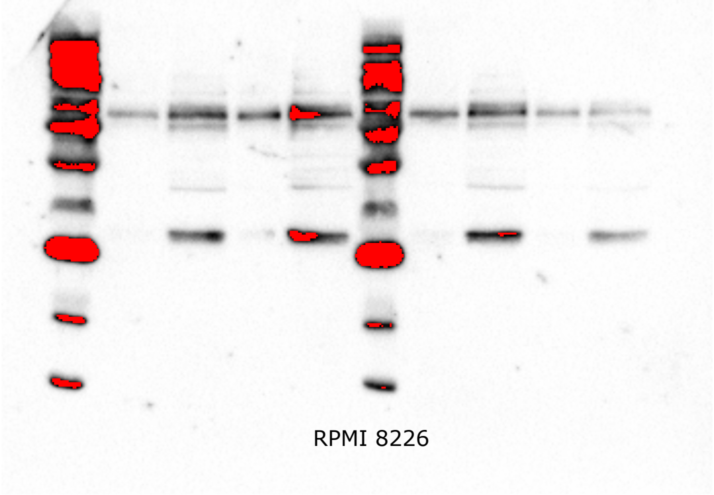

B-actin

Right blot used in manuscript, lanes 7 and 8

Loading order:
Control - Daratumumab

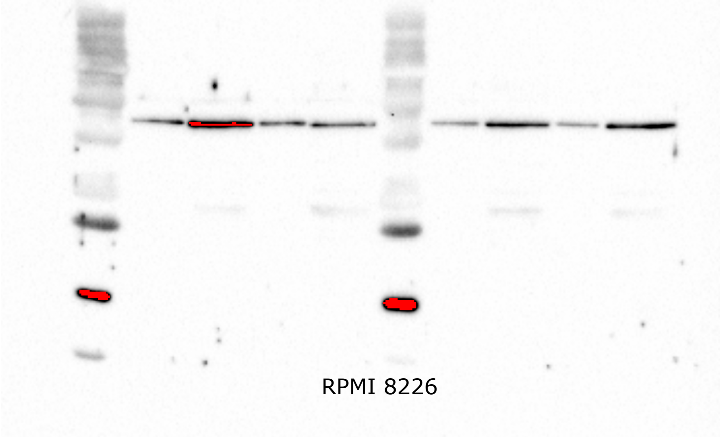

Supplement: Supplementary file 1 [file cancers-13-01662-s001.pdf]
